# Preparation of macroporous transition metal hydroxide monoliths via a sol-gel process accompanied by phase separation

**DOI:** 10.1038/s41598-020-61195-9

**Published:** 2020-03-09

**Authors:** Fu Liu, Daoyan Feng, Hui Yang, Xingzhong Guo

**Affiliations:** 1 State Key Laboratory of Silicon Materials, School of Materials Science and Engineering, Zhejiang University, Hangzhou, 310027 PR China; 2 Zhejiang-California International Nanosystems Institute, Zhejiang University Hangzhou, 310058 PR China

**Keywords:** Environmental monitoring, Inorganic chemistry, Chemical engineering, Porous materials

## Abstract

Three-dimensional transition metal hydroxide monoliths were facilely fabricated by a sol-gel process accompanied by phase separation in the presence of polyacrylic acid (PAA) and propylene oxide (PO). In the typical ZnCl_2_-PAA-PO system, PAA is used as a phase separation inducer as well as a framework former to control the phase separation and the formation of macrostructures, whereas PO works as a proton scavenger to initiate the gelation of the system and freeze the macrostructures. Appropriate amount of PAA, PO and solvents allow the formation of zinc (Zn) hydroxide monolith with cocontinuous skeletons and interconnected macropores, and the construction mechanism and characteristics of macrostructure are also investigated. The resultant dried gels are amorphous Zn hydroxide monolith with a narrow macropore size distribution (~1 μm). This approach is further used to successfully prepare macroporous single or binary composite transition metal hydroxide monoliths.

## Introduction

Transition metal hydroxides have special functional groups and distinctive structures, and possess high adsorption capacity to polar substances and excellent performance in adsorbing harmful and contaminant materials^1^, water splitting^2^ and supercapacitor^3^ in the field of energy^4^, catalysis^5^ and environment, etc. Up to now, two dimensional structural transition metal hydroxides such as thin film, layer^6,7^ or sheet^2^, have been synthesized by numerous techniques. Since porous materials have attached more and more attention, the synthesis of porous transition metal hydroxides seems to be a new research direction of these materials.

In recent years, transition metal oxide monoliths with macroporous structure have been realized successfully in several species using a sol-gel process accompanied by phase separation^8–10^. In the process, the inorganic salts or metal alkoxides hydrolyze and polycondense to form a uniform sol, then the formed colloidal particles are slowly polymerized to form a skeleton of a 3-dimensional framework structure to finish the gel transformation, and the obtained gel undergoes aging, drying and heat treatment processes to form the desired oxide materials^11,12^. The traditional sol-gel method uses alkoxide as a precursor, and the preparation process is relatively mature. The sol-gel process accompanied by phase separation combines the phase separation induced by spinodal decomposition with the sol-gel transition together, and can “freeze” or “fix” the transition state structure in the phase separation process through the sol-gel transition. The macroporous structure is introduced into the morphology of the material. Gash *et al*.^13^ used sol-gel method to prepare iron-based aerogel monoliths with inorganic salts (Fe(NO_3_)_3_∙9H_2_O, FeCl_3_∙6H_2_O) as precursors, polar molecules such as ethanol and water as solvents. During the reaction, the epoxide undergoes an irreversible ring-opening reaction, which controls the pH of the system to induce the conversion of the sol into the gel system. During the transformation, the primary particles of the gel do not agglomerate to form a precipitate, so that the framework phase and solvent phase form a “block” and remain to obtain monolithic material. This method by using inorganic salts as precursors has been carried out and confirmed in the preparation of transition metal oxide^8,14–19^, metal oxide^10,20,21^ and rare earth metal oxide^22–24^ monolithic materials.

In this work, we report a facile preparation of transition metal hydroxide monoliths using epoxide-induced sol-gel process accompanied by phase separation. And the effects of polyacrylic acid (PAA), solvent, propylene oxide (PO) and inorganic salt precursor on the gel morphology and the formation mechanism of macrostructure have been investigated in detail. In addition, this approach is also extended to prepare other macroporous single and binary composite transition metal hydroxide monoliths.

## Experimental

### Materials

Zinc chloride anhydrous (ZnCl_2_), cobalt chloride hexahydrate (CoCl_2_·6H_2_O), nickel chloride hexahydrate (NiCl_2_·6H_2_O), manganese chloride (MnCl_2_·4H_2_O), iron chloride tetrahydrate (FeCl_2_·4H_2_O) were all purchased from Aladdin. Poly (acrylic acid) (PAA, 35 wt% in water, average molecular weight of 100,000) was supplied from Sigma-Aldrich. Glycerol (C_3_H_8_O_3_), propylene oxide (PO), ethanol absolute (C_2_H_6_O) were obtained fromSinopharm Chemical Reagent Co., Ltd.

### Preparation of macroporous zinc hydroxide monolith

A typical preparation procedure is as follows. Firstly, 1.2 mL of distilled water and 2.4 mL of glycerol were mixed in a glass tube with vigorous stirring, then 1.34 g ZnCl_2_ was added and stirred to completely dissolve. After that, 3.2 g of PAA was added slowly and the mixture was stirred continuously to form a clear and transparent solution. 2.2 ml PO was added dropwise in the mixture following by stirring for 5 min. The glass tube with homogeneous sol was ultrasonicated for 10 s to eliminate the gas and was kept at 60 °C for gelation and aging for 12 h. The obtained wet gel underwent solvent replacement with ethanol for three times and then was dried at 60 °C for 2 days. Other single and binary composite transition metal hydroxide monolith samples were produced by only changing corresponding reagents and residual aspects were unchanged.

### Characterization

Microstructure and morphology of the synthesized hydroxide monolith were observed with a scanning electron microscope (SEM, SU-70, Hitachi Ltd., Japan). The element distribution was examined by energy-dispersive X-ray spectroscopy (EDS) attached to the SEM. The phase composition was characterized by powder X-ray diffraction (XRD, PANalytical B.V., Holland). Chemical bonding information was examined by Fourier-transform infrared spectroscopy (FTIR, Nicolet 5700, Thermo Fisher Co., USA). Thermogravimetry-differential thermal analysis (TG-DTA, SDT-Q600, TA Instrument, USA) of the xerogels were executed at a heating rate of 5 °C min^−1^.To determine the composition and coordination state of the xerogels, X-ray photoelectron spectroscopy (XPS) studies were carried out on a Thermo Scientific K-Alpha + X-ray Photoelectron Spectrometer (XPS). Macropore size distribution of the obtained samples was evaluated by mercury intrusion porosimetry (AutoPore IV 9510, Micromeritics Ins. Ltd., USA).

## Results and Discussion

### Formation and control of macrostructure for Zn Hydroxides

#### Effect of PAA on macrostructure

**Figure 1:**
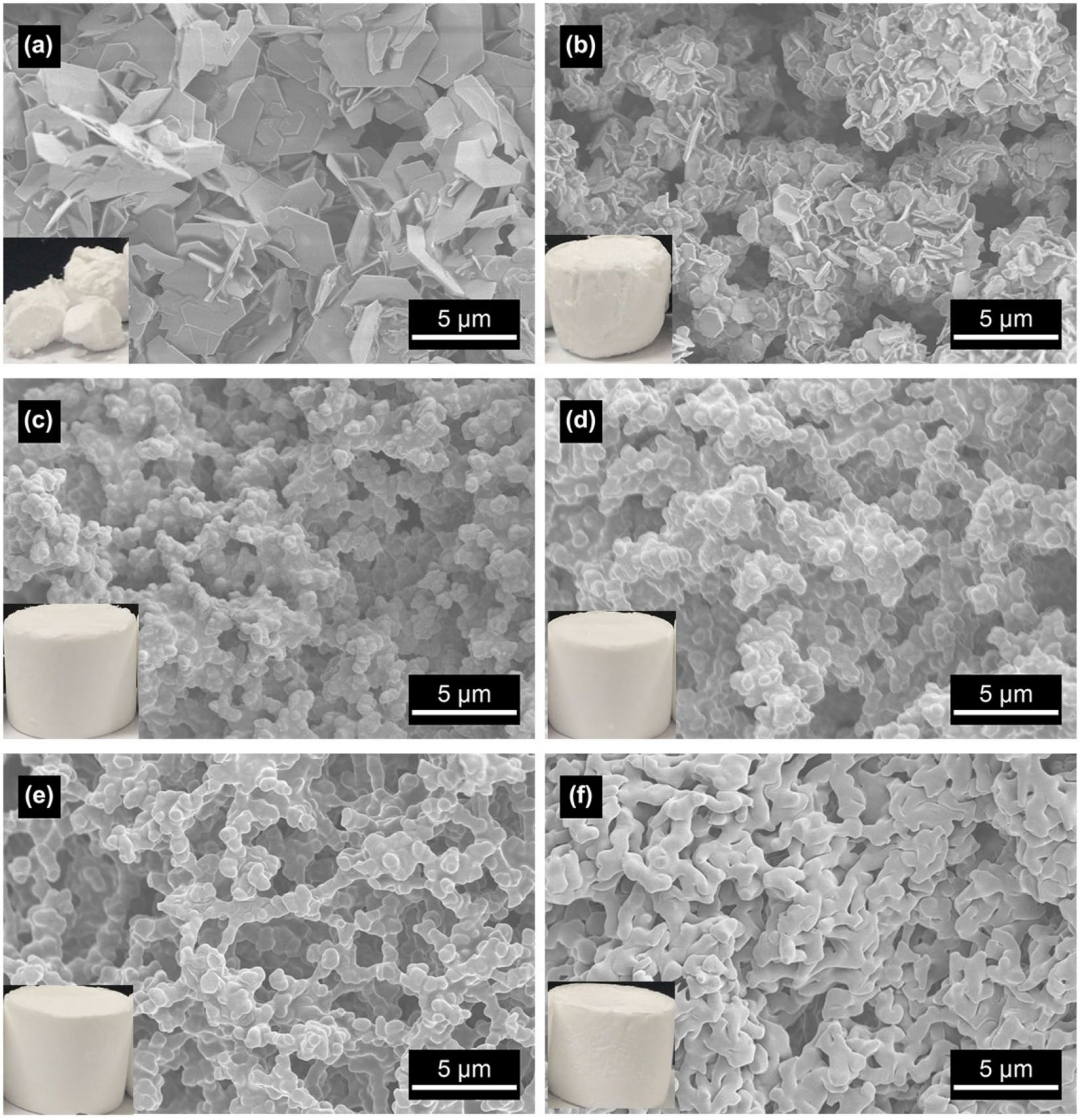
SEM images of xerogel samples with varied PAA amounts: (**a**) 0 g, (**b**) 0.8 g, (**c**) 1.6 g, (**d**) 2.4 g, (**e**) 3.2 g and (**f**) 4.0 g.

In this system, PAA is added as phase separation inducer to induce the phase separation of the system. Owing to the capillary force, the sample without PAA is fragment after drying, while the samples with PAA do not change significantly after drying, as shown in Fig. 1. It indicates that PAA has an effect of relieving capillary force and enhancing the strength of the skeletons. From Fig. 1a, the Zn hydroxide without PAA exists as aggregated nanosheets. With the increase of PAA content, the size of nanosheets decreases gradually, whereas a three-dimensional macrostructure with a cocontinuous skeletons and interconnected macropores forms successively, as shown in Fig. 1b~1d. However, with an excessive amount of PAA, the macrostucture becomes a cluster/bundle precipitate, which is caused by the flocculation of the inorganic salt precursor with the PAA molecular chain (Fig. 1f). When the PAA amount is 3.2 g, a smooth Zn hydroxide monolith with an exquisite three-dimensional macrostructure is obtained (Fig. 1e).

Further comparing with the two samples without PAA (Fig. 1a) and with 3.2 g PAA (Fig. 1e), it is found that PAA also has an important effect on the microstructure of Zn hydroxide. Without PAA, the gel particles are crystalline nanosheet aggregates, indicating the occurrence of precipitation reaction. After the addition of PAA, the crystalline nanosheets are replaced by amorphous cocontinuous skeletons. It specifies that PAA not only controls phase separation of the system to form the macrostructure but also restrains the precipitation reaction to produce the crystalline nanosheets. And the mechanism of PAA on phase separation and precipitation will be discussed in next section.

#### Effect of PO on macrostructure

**Figure 2:**
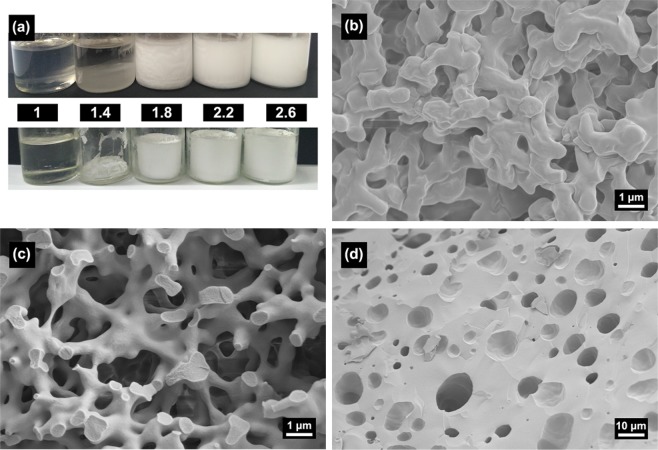
(**a**) sol-gel transform in 30 min (up) and appearance of typical xerogel samples (down) and SEM images of xerogel samples with different PO contents: (**b**)1.8 mL, (**c**) 2.2 mL, (**d**) 2.6 mL.

In this work, PO acts as a gelation promotor, after the addition of PO, the sol-gel transition was quickly completed within 30 min, and the gelation time decreased with the increase of PO amount. Figure 2a shows the actual photos of the samples with different PO contents in the glass tube. When the amount of PO is 1.0 mL, the gelation does not occur, and it is a clear and transparent sol. When the PO rises to 1.4 mL, the system is separated into two layers, which indicates that insufficient PO becomes a precipitant to precipitate part of the sol components. As the amount of PO increases to 1.8, 2.2 and 2.6 mL, the system is completed gelatinized at 15, 9 and 7 min, respectively.

The effect of PO content on the macrostructure of the Zn hydroxide monoliths is shown in Fig. 2b~2d. When the amount of PO is1.8 mL, the skeletons are composed of rough particles (Fig. 2b). An excessive PO (2.6 mL) results in isolated and very large pores (Fig. 2d). When an appropriate amount of PO (2.2 mL) is added, the macrostructure with cocontinuous skeletons and interconnected macropores can be obtained (Fig. 2c). It can be interpreted that different PO amount corresponds to different periods of phase separation process. Less amount of PO makes the sol-gel transition occur at the end of phase separation, and the structure has undergone slight Ostwald ripening. And more amount of PO, on the contrary, makes the sol-gel transition take place at the early stage of phase separation, and phase separated locally in the system and the interface between the two phases is not obvious. By adding an appropriate PO amount, the system can get the sol-gel transition match well with phase separation and produces an ideal cocontinuous macrostructure.

#### Effect of solvents on macorstructure

The choice of solvent is very significant in preparing porous monoliths by sol-gel process accompanied by phase separation. The solvent mainly exists in the solvent phase after phase separation, surrounding the framework phase, and it will be evaporated during the subsequent drying process and forms voids around the original skeleton phase. Therefore, the size and distribution of the pores are mainly determined by the species and amount of the solvent.

**Figure 3:**
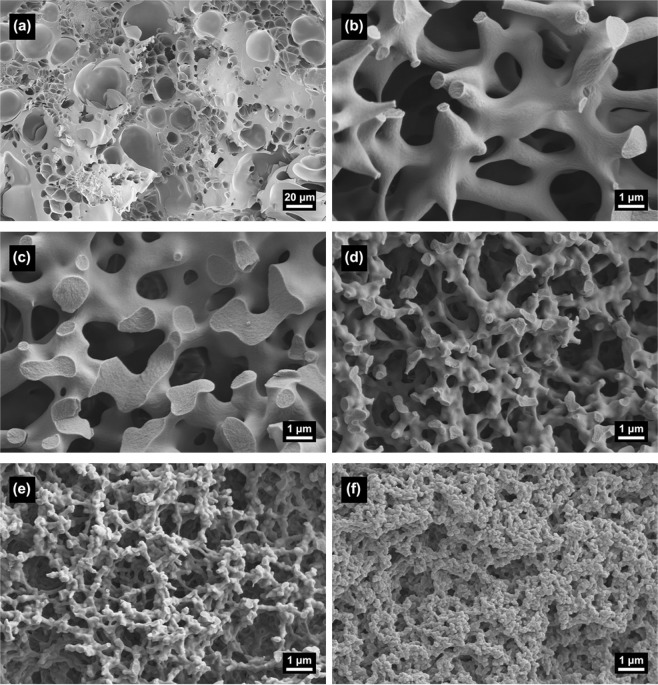
SEM images of xerogel samples with varied amounts of solvent, (**a**) H:G = 2:1.6, (**b**) H:G = 1.6:2.0, (**c**) H:G = 1.2:2.4, (**d**) H:G = 0.8:2.8, (**e**) H:G = 0.4:3.2and (**f**) H:G = 0:3.6, respectively.

Figure 3 shows the microstructure of macroporous zinc hydroxide monoliths prepared with different solvent amounts. When the ratio of water to glycerol in the solvent system is 2:1.6, it is difficult to form a cocontinuous macrostructure due to a large number of aqueous phases (Fig. 3a). As the glycerol content increases, the morphology of the section transits to a cocontinuous macrostructure (Fig. 3b), and finally a porous structure with a uniform pore size distribution (Fig. 3c) can be achieved when the ratio of water to glycerol is 1.2:2.4. With the further increase of glycerol content, the cocontinuous macrostructure still maintains (Fig. 3d~f), but the macropores become small.

#### Effect of precursor on macrostructure

**Figure 4:**
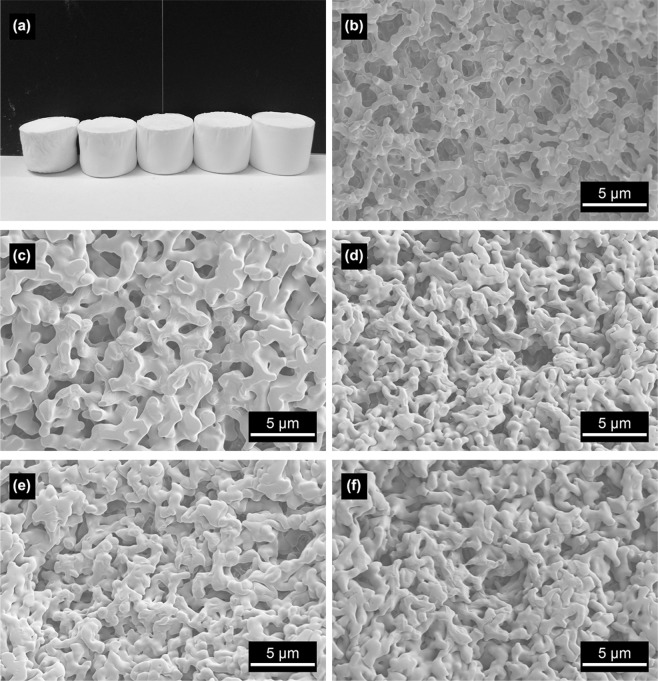
(**a**) Appearance of typical xerogel sample and SEM images of xerogel samples with a varied amount of precursors added; (**b**) 1.22 g, (**c**) 1.34 g, (**d**) 1.46 g, (**e**) 1.58 g and (**f**) 1.70 g, respectively.

The amount of inorganic salt precursor determines the number of particles that can be polymerized on the same volume of the framework, and further affects the size of the framework. As shown in Fig. 4a, the Zn hydroxide xerogels with different precursor amounts have a certain size and its shape can be controlled during the gel process. SEM images (Fig. 4b~f) show the morphological change of Zn hydroxide xerogel with different precursor amounts. When the amount of inorganic salt is few, a cocontinuous macrostructure can be obtained (Fig. 4b). As the amount of precursor increases, the cocontinuous skeletons become denser and larger, and a perfect macrostructure can be achieved (Fig. 4c). With the further increase of precursor amount, the morphology and monolithic shape are basically kept, while the macrostructure becomes gradually worse with the decrease of macropore size and the disappearance of cocontinuous skeletons (Fig. 4d,e). Inorganic metal salts undergo hydrolysis to form hydrated metal ions complex with water molecules and further polycondense to form particles. The particles will combine with the PAA molecular chain to form the skeletons, eventually form a porous gel framework. More inorganic salts precursor results in more particles, making the skeleton denser eventually.

### Construction mechanism of macrostructure for transition metal hydroxide monolith

As reported, the thermodynamic process of mixed solutions system containing polymer is described by the Flory-Huggins equation^25–27^. The free energy of the mixed system changes as shown in the following equation:

1 $$\Delta G=-\,T\Delta S+\Delta H=RT\left(\frac{{\varphi }_{1}}{{P}_{1}}\,\mathrm{ln}\,{\varphi }_{1}+\frac{{\varphi }_{2}}{{P}_{2}}\,\mathrm{ln}\,{\varphi }_{2}+{\chi }_{12}{\varphi }_{1}{\varphi }_{2}\right)$$

Wherein, *φ*_*i*_ and *P*_*i*_ (*i* = 1, 2) are the volume fraction in the mixed system and the degree of polymerization of the component respectively, and *χ*_12_ is the interface parameter of the two components. The first two terms in equation correspond to the entropy change of the system, and the last one contributes to the enthalpy change of the system. As seen from the equation, increasing the polymerization degree of any component will decrease the entropy change (Δ*S*). The increase of interfacial parameter between the mixed components can accrete the enthalpy change (Δ*H*). Therefore, the entropy change (Δ*S*) of the system decreases, and the enthalpy change (Δ*H*) increases, resulting in the Gibbs free energy (Δ*G*) of the mixed binary system gradually changing from a negative value to a positive value, meantime the mixed system changes to an unstable state. When the Gibbs free energy is greater than zero, the incompatibility of the mixed system reaches a critical point, and the mixed system begins to phase separate.

In this real transition metal sol-gel system, the inorganic metal salt generally presents in the form of hydrated metal ions complexed with water molecules in solution:

2 $${{\rm{M}}}^{n+}+x{{\rm{H}}}_{2}{\rm{O}}\rightleftharpoons {[{\rm{M}}{({{\rm{H}}}_{2}{\rm{O}})}_{x}]}^{n+}$$

M^*n*+^ and *x* represent a metal ion and a coordination number of a hydrated ion, respectively. The hydrated zinc ion ([Zn(H_2_O)_6_]^2+^) can undergo further multistage dissociation equilibrium in aqueous solution, releasing hydrogen ions (formula 3). The hydrated zinc ion ([Zn(H_2_O)_6_]^2+^) and [Zn(OH)(H_2_O)_5_]^+^ form a uniformly stable sol with PAA, glycerol and water. Because of the strong hydrogen bond between the hydrated ions and PAA, the polymerization degree (*P*_*i*_) of the systems increases, resulting in the decrease of the entropy change (Δ*S*) and further increment of Gibbs free energy (Δ*G*). When Δ*G* becomes a positive value, the phase separation will occur.

3 $${[{\rm{Zn}}{({{\rm{H}}}_{2}{\rm{O}})}_{6}]}^{2+}\rightleftharpoons {[{\rm{Zn}}({\rm{OH}}){({{\rm{H}}}_{2}{\rm{O}})}_{5}]}^{+}+{{\rm{H}}}_{3}{{\rm{O}}}^{+}$$

4

**Figure 5:**
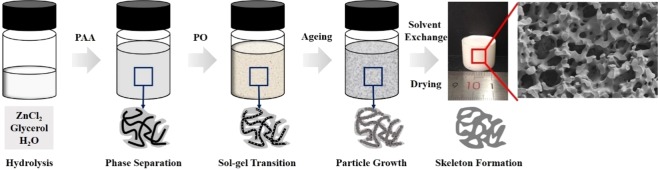
Schematic illustration of macrostructure formation for Zn hydroxide monoliths.

With the addition of epoxide (PO), the H^+^ions produced by the hydrolysis of zinc ions will be consumed through irreversible ring-opening reaction caused by the nucleophilic induction of Cl^−^ (formula 4), the equilibrium will be shifted to the right, and the pH value of the system will be gradually increased, accelerating the polycondensation and crosslinking between [Zn(OH)(H_2_O)_5_]^+^ (forming Zn-O-Zn chemical bond) to form particles, further realizing the conversion of sol-gel^13^. After the sol-gel transition, the transition state of the system phase separation can be “fixed”, and a cocontinuous macrostructure can be obtained after drying. The cooperative control of phase separation and sol-gel transition becomes very important. If the sol-gel transition occurs at the end of phase separation, the system will obtain the Ostwald ripening structure. Correspondingly, if it occurs in the initial stage of phase separation, the morphology of the initial phase separation state will be obtained. Only when the sol-gel transition is equivalent to the phase separation process, an ideal cocontinuous macrostructure can be achieved, as shown in Fig. 1e. Subsequently, since the particles formed during the sol-gel conversion process have a strong hydrogen bond with the PAA, the particles grow along the entire PAA molecular chain and randomly bind together, eventually form a porous gel framework throughout the system, as illustrated in Fig. 5. Furthermore, the carboxyl of PAA complexes with the particles, restraining the coprecipitation of the particles. Thence the crystalline nanosheet aggregates form in the sample without PAA appears, as shown in Fig. 1a.

**Figure 6:**
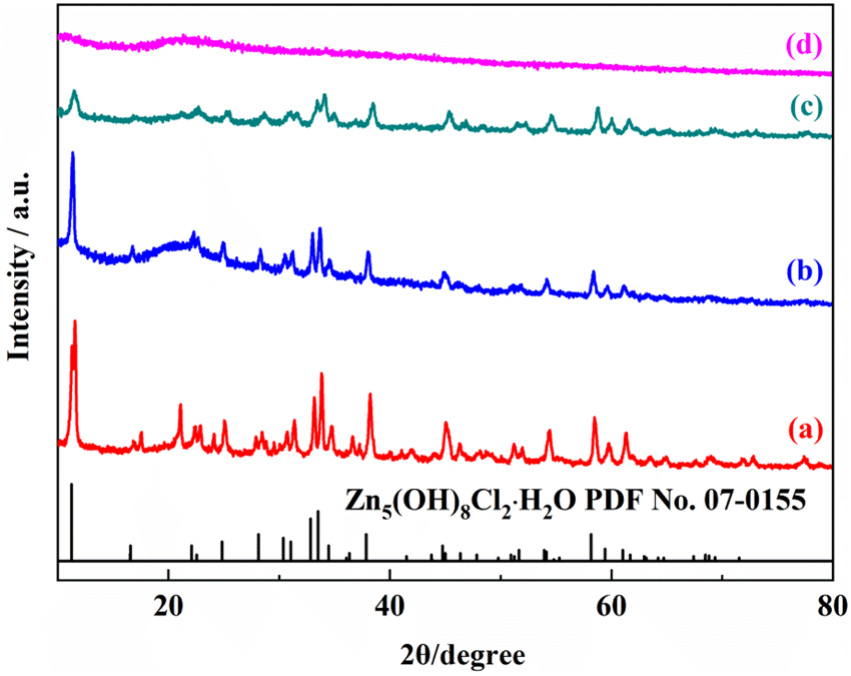
Typical XRD curves of samples with different PAA amount (**a**) 0 g, (**b**) 0.8 g, (**c**) 1.6 g, (**d**) 2.4 g.

The XRD patterns in Fig. 6 furtherly confirm the function of PAA in impeding precipitation and crystallization. Without PAA, there are sharp diffraction peaks in the dried xerogel, confirming the formation of complete crystalline phase. The diffraction peaks correspond to Zn_5_(OH)_8_Cl_2_·H_2_O (PDF No. 07-0155). As the amount of PAA increases, the intensity of the diffraction peaks becomes weaker. When the amount increases to 2.4 g, the diffraction peak completely disappears and the crystalline phase transits to amorphous Zn hydroxide, which indicates that PAA has a function of inhibiting the crystallization and precipitation of the products. It confirms that PAA not only plays a role in inducing phase separation of the system but preventing the formed particles from colliding with each other and precipitation.

**Figure 7:**
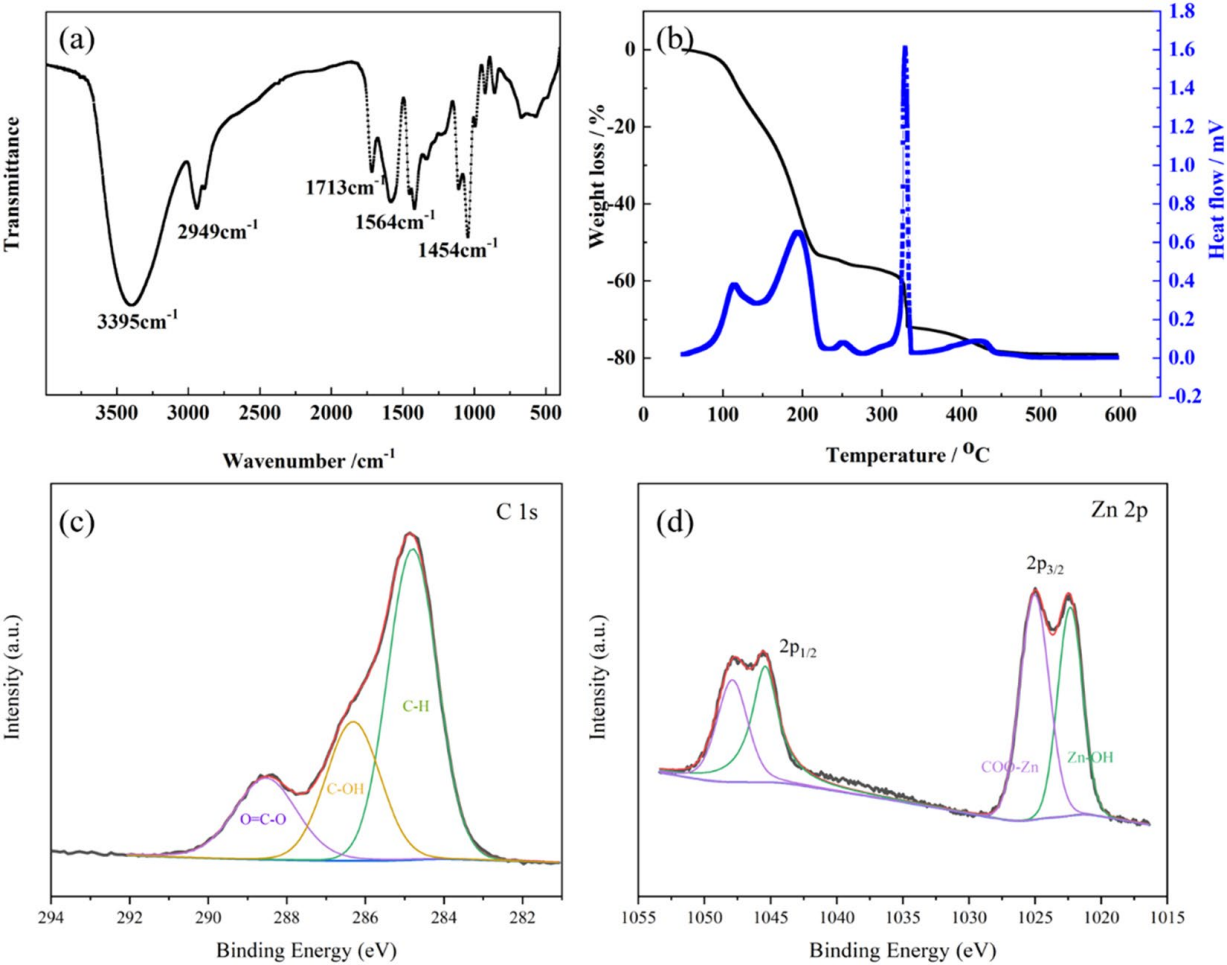
(**a**) FTIR profile of Zn hydroxide and (**b**) Typical TG/DTA curves, XPS spectra of typical xerogel (**c**) C1s, (**d**) Zn 2p.

In order to further characterize the distribution of PAA, the sample was characterized by infrared and thermogravimetry, as shown in Fig. 7. Infrared absorption peaks at 1454, 1564, 1713, 2949 and 3395 cm^−1^ are assigned to PAA (Fig. 6a)^28^, which indicates that PAA exists in the solid phase. And PAA is preferentially distributed in the solid phase since -COOH of PAA can interact with -OH of Zn hydroxide through hydrogen bonding. The thermogravimetric curve at 90 °C has a 2% mass loss and endothermic effect, which is attributed to the evaporation of residual solvent and water adsorbed on the surface (Fig. 6b). When the temperature rises to about 115 °C, there is about 3% mass loss and exothermic effect, which is contributed by the pyrolysis heat of chlorohydrin, the ring-opening reaction product. A strong exothermic peak at 200 °C is attributed to the pyrolysis of glycerol, indicating that glycerol also exists in the solid phase^29^. The mass loss and exothermic peak at temperatures up to 330 °C correspond to the thermal liberation effect of PAA pyrolysis. The infrared and thermogravimetric data indicate that PAA and glycerol are preferentially distributed in the solid phase after phase separation, performing as framework former. In the XPS spectrum of C 1 s (Fig. 7c), the peaks at 284.7, 286.2 and 288.4 eV can be assigned to C-H, C-OH and O=C-O, respectively, indicating the distribution of PAA and glycerol on the surface of the skeletons^30^. And two peaks of COO-Zn and Zn-OH at 1025.1 and 1022.3 eV in Fig. 7d prove the formation of zinc hydroxide and reaction between Zn^2+^ and carboxyl in PAA, revealing the good combination of PAA and hydrolyzed particles.

**Figure 8:**
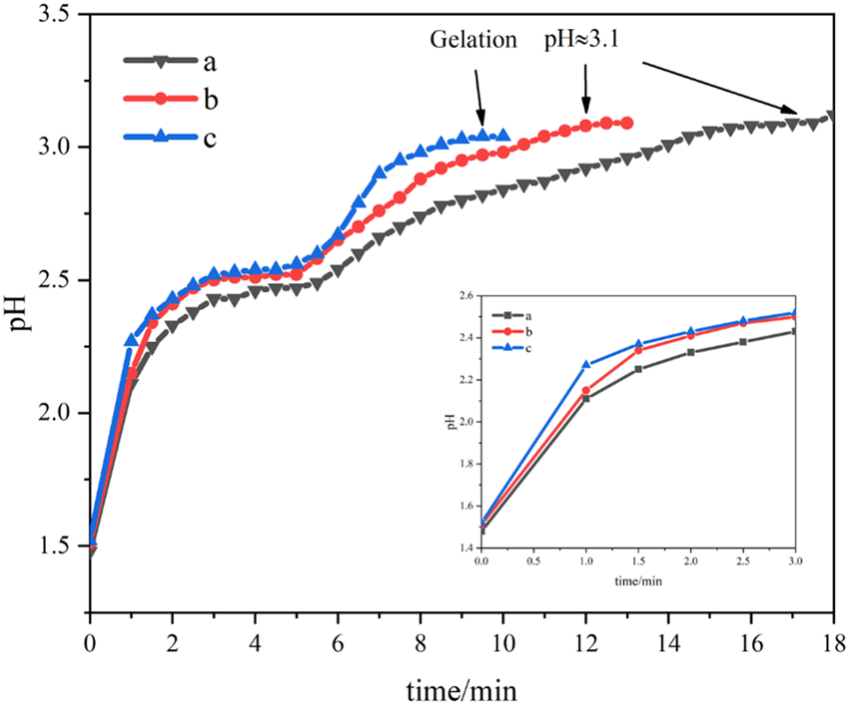
Time evolutions of pH value after addition of different amounts of PO (**a**) 1.8 mL, (**b**) 2.2 mL, (**c**) 2.6 mL.

As we know, the sol-gel transition caused by the increase of pH in the system has a significant effect on the transition state structure resulting from the “fixed” phase separation process^31^. The pH of the system rises through the process that the H^+^ ions are captured by the irreversible ring-opening reaction of PO (formula 4), which controls the sol-gel conversion in the phase separation process. The amount of PO directly affects the rising rate of pH and furtherly the gelation time. The relationship between pH and time of different PO content is shown in Fig. 8. The pH of the system rises rapidly soon after the addition of PO, indicating the irreversible ring-opening reaction. The pH rises faster with more PO content, and the gelation time of the systems with PO contents of 1.8, 2.2 and 2.6 mL are about 15, 9 and 7 min respectively. When the pH of the system is about 3.1, the gelation occurs for three solutions. Different gelation time corresponds to various phase separation degrees, and controllable phase-separated structures can be obtained (Fig. 2).

**Figure 9:**
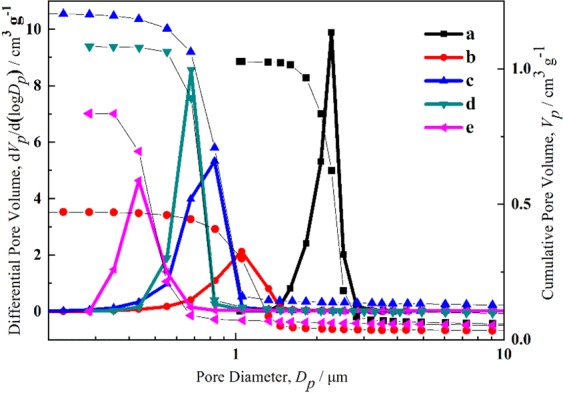
Mercury porosimetry results of the xerogel samples with varied amounts of solvent (**a**) H:G = 1.6:2.0, (**b**) H:G = 1.2:2.4, (**c**) H:G = 0.8:2.8, (**d**) H:G = 0.4:3.2, (**e**) H:G = 0:3.6.

The ratio of H_2_O and glycerol determines the hydrolysis of precursor, phase-separation tendency and the proportion of separated liquid-solid phases, and further controls the size of solid skeletons. In the system with a larger amount of H_2_O, the hydrolysis reaction of Zn^2+^ is more strongly promoted to increase the number of hydroxyl groups on the surface of the oligomers. Because of the strong hydrogen bond and complexation between hydroxyl groups and PAA molecular chains, the interface parameter (*χ*_12_) is smaller and Gibbs free energy (Δ*G*) decreases, the phase-separation tendency becomes weaker, and the finer skeletons are obtained as a result of the later onset of phase separation. On the contrary, larger glycerol content inhibits the hydrolysis process, leading to an earlier phase separation process and coarser skeletons, as shown in Fig. 3. The median macropore size decreases slightly with the decrease of H_2_O and corresponding increase of glycerol in the same solvent content, as shown in Fig. 9. All the samples possess sharp and narrow macropore size distribution, reflecting the macrostructure formed via the spinodal decomposition. The macropore size of typical xerogel with solvent ratio as H:G = 1.2:2.4 is distributed roughly between 0.5 and 1.5 μm, and the median macropore size is 1.0 μm.

**Figure 10:**
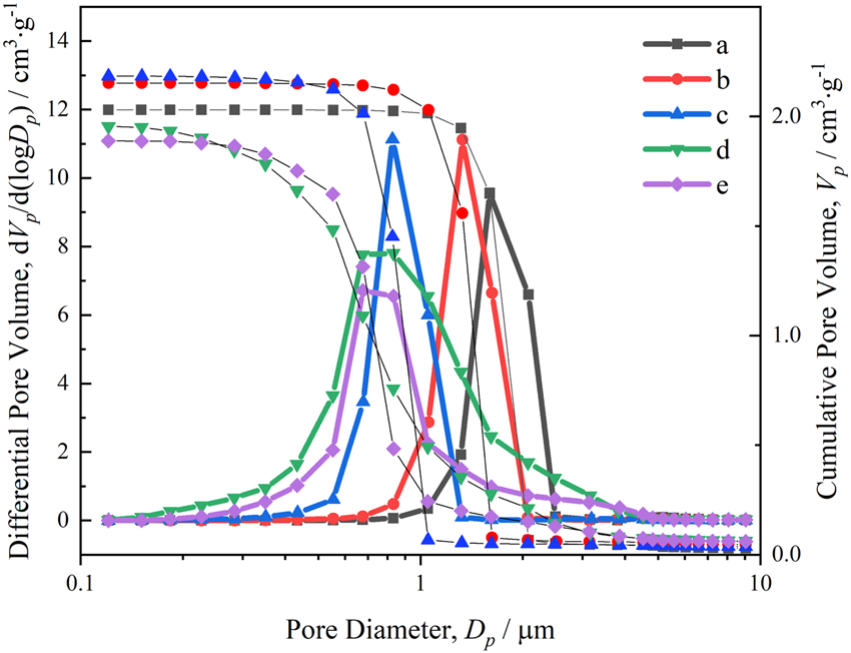
Mercury porosimetry results of the xerogel samples with varied amounts of ZnCl_2_ (**a**) 1.22 g, (**b**) 1.34 g, (**c**) 1.46 g, (**d**) 1.58 g, (**e**) 1.70 g.

The metal ions undergo the hydrolysis to form the hydrated metal ions and further polycondense to form the particles. It means that high precursor content produces more particles. During the polymerization and aging, the leftover hydrated ions gradually combine with the particles in skeletons formed previously to densify and coarsen the skeletons, as shown in Fig. 4. Correspondingly, the median macropore size decreases with the increase of ZnCl_2_ amount, as shown in Fig. 10. The macropore size distribution expands with more precursors, indicating the uneven macropore formation caused by uneven precipitation of the particles. Suitable precursor content can control the macropore size of monolith and the densification of skeletons.

### Preparation of single and binary composite transition metal hydroxide monoliths

For high-valence elements (Si, Ti, etc.), alkoxides can be selected as precursors. High-valence inorganic salt elements exist in the form of hydrated ions in aqueous solution, which exhibits strong acidity and can undergo a multi-stage hydrolysis reaction process and sol-gel conversion^29,32^.The high valence state of those alkoxides results in a high degree of freedom and easy formation of particles, and the particles further interconnect to form three-dimensional frameworks.The alkoxide precursors corresponding to the low valent elements (Mn^2+^, Fe^2+^, Co^2+^, Ni^2+^, Zn^2+^) are more active, sensitive to humidity and are difficult to effectively control hydrolysis and polycondensation.Because of their low valence state, the hydrolysis activity is weak and the degree of freedom is small^16,33^, and it is difficult to form a three-dimensional framework of particles to realize sol-gel transition.

**Figure 11:**
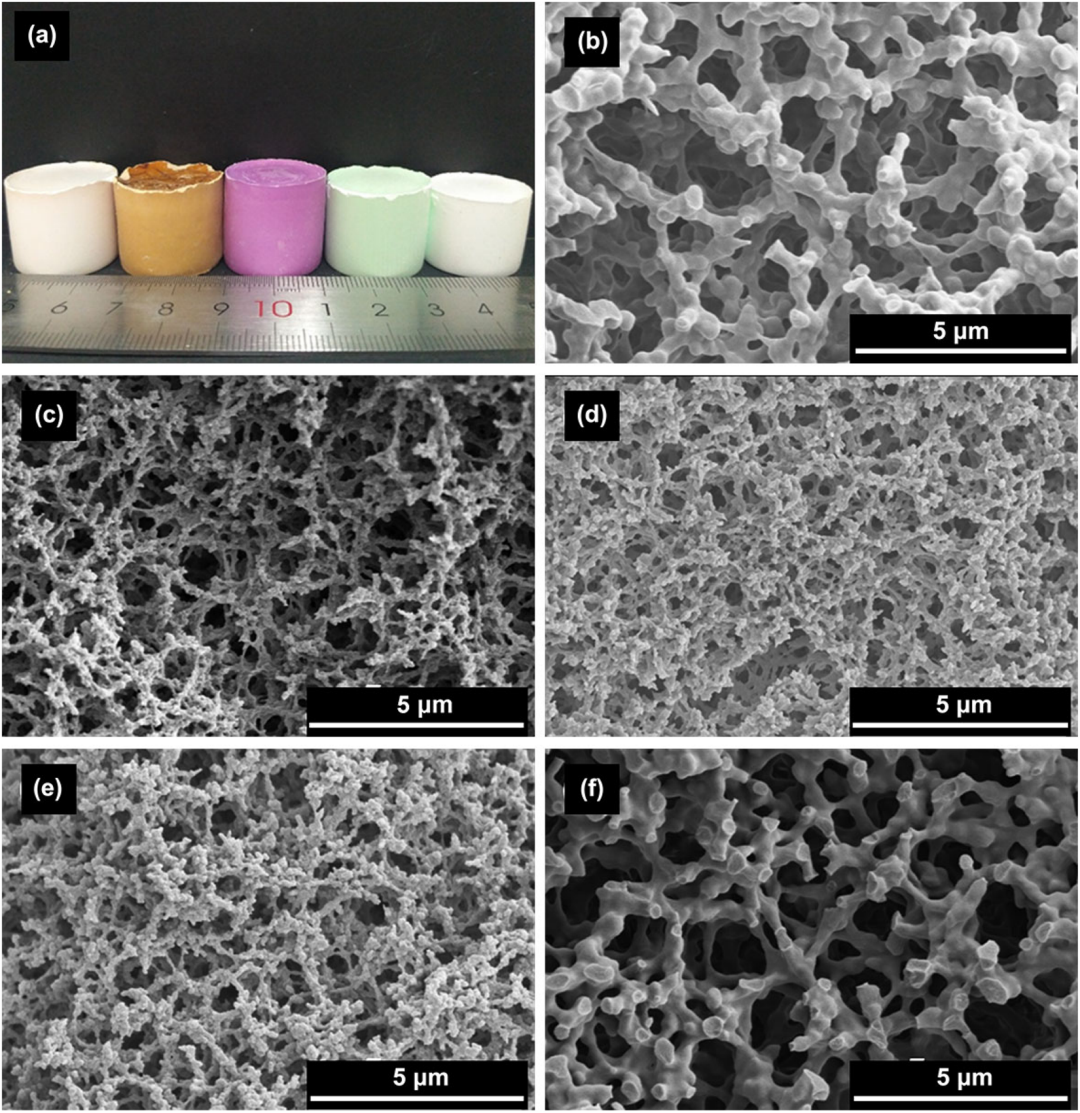
Appearance of typical xerogel monoliths (**a**) and microstructures of transition metal hydroxide monoliths with different precursors (**b**) Mn^2+^, (**c**) Fe^2+^, (**d**) Co^2+^, (**e**) Ni^2+^ and (**f**) Zn^2+^, respectively.

In the above ZnCl_2_-PAA-PO system, we have developed a facile approach to prepare zinc hydroxide monoliths by using epoxide-induced sol-gel process accompanied by PAA-induced phase separation. In order to verify the universality of this approach, the preparation of other single transition metal (such as Mn^2+^, Fe^2+^, Co^2+^, Ni^2+^and Zn^2+^) hydroxide monoliths are also studied by means of this approach. It is noted that with the constant addition amounts of inorganic salt precursor, the macrostructure with cocontinuous skeletons and interconnected macropores can form in all the samples through the typical preparing process, as shown in Fig. 11, which confirms that this approach has universality for preparing macroporous transition metal hydroxide monoliths.

**Figure 12:**
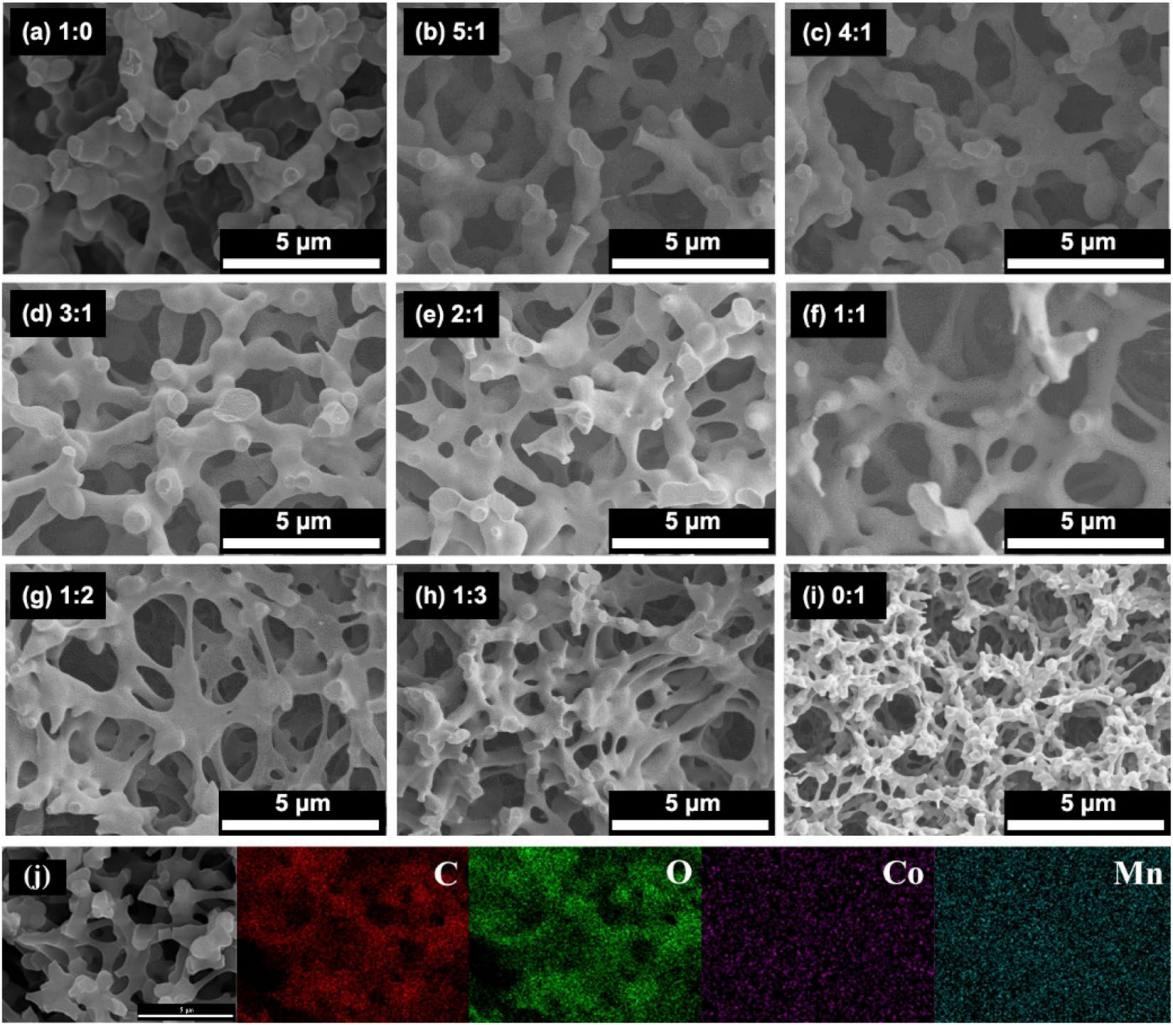
SEM images of xerogel samples with varied ratios of Mn and Co precursors added (**a**)Mn: Co = 1:0, (**b**) Mn: Co = 5:1, (**c**) Mn: Co = 4:1, (**d**) Mn: Co = 3:1, (**e**) Mn: Co = 2:1, (**f**) Mn: Co = 1:1, (**g**) Mn: Co = 1:2, (**h**) Mn: Co = 1:3, (i) Mn: Co = 0:1, respectively and (**j**) SEM and the corresponding EDS elemental mappings of different elements of sample with Mn: Co = 2:1.

**Figure 13:**
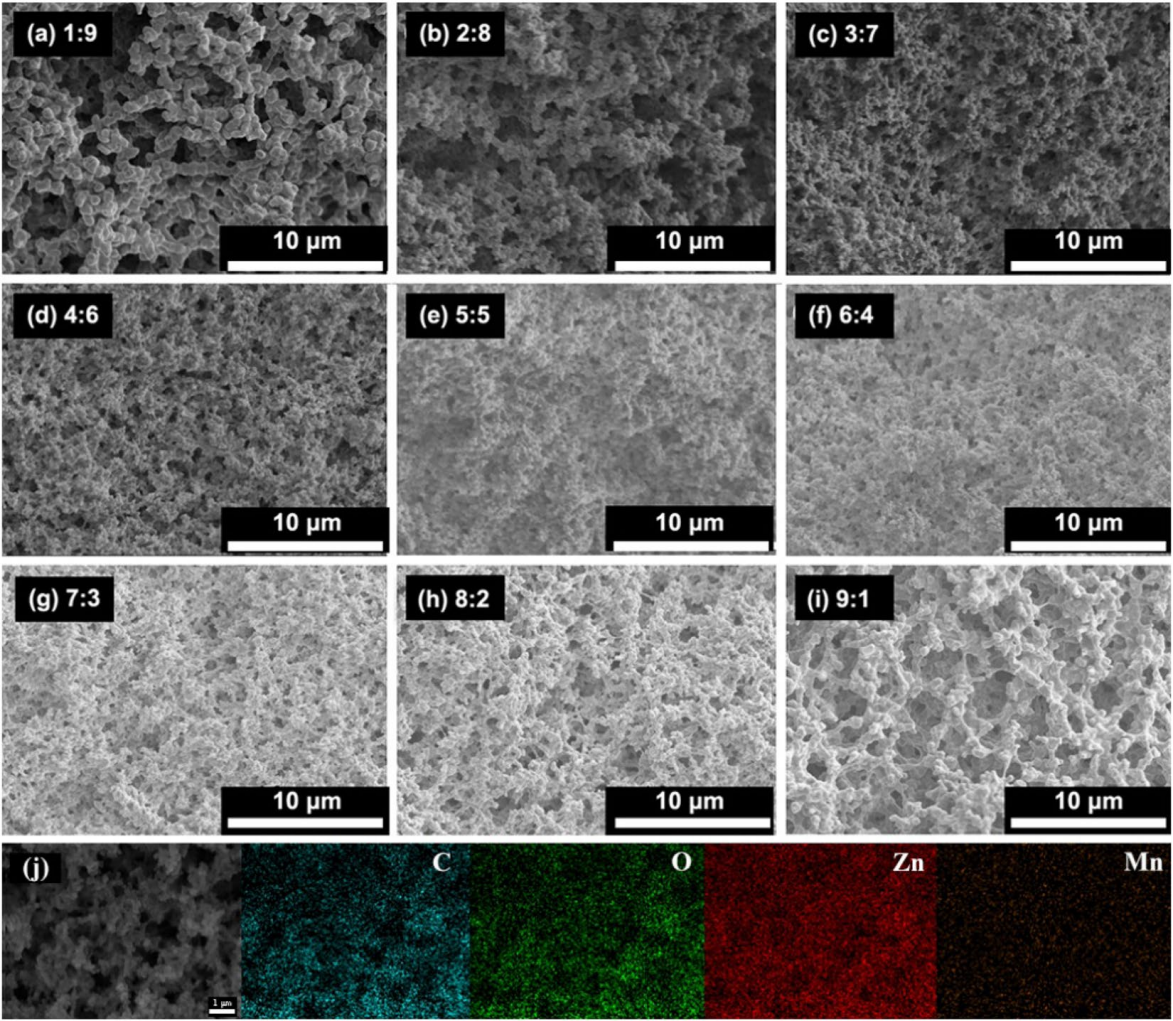
SEM images of xerogel samples with varied ratios of Mn and Co precursors added (**a**) Zn: Mn = 1:9, (**b**) Zn: Mn = 2:8, (**c**) Zn: Mn = 3:7, (**d**) Zn: Mn = 4:6, (**e**) Zn: Mn = 5:5, (**f**) Zn: Mn = 6:4, (**g**) Zn: Mn = 7:3, (**h**) Zn: Mn = 8:2, and (**i**) Zn: Mn = 9:1, respectively and (**j**) SEM and the corresponding EDS elemental mappings of different elements of sample with Zn: Mn = 9:1.

Similarly, this approach was also used to prepare binary composite transition metals (such as Mn^2+^ and Co^2+^, Zn^2+^and Mn^2+^) hydroxide monoliths. In the preparation, the addition amounts of PAA, PO and the ratio of water to glycerol are constant, and only the inorganic salt precursor is changed. Figures 12 and 13 show the microstructure obtained by changing the molar ratios of Mn and Co and Zn and Mn precursor salts, respectively. It can be seen from Fig. 12 that a cocontinuous macrostructure can be obtained regardless of the ratio of the two precursors. Compared with the structure of pure Mn and pure Co system, the higher the content of the precursor, the closer the macrostructure is to which system. The skeletons of the pure Mn system is relatively coarse (Fig. 12a), which is relatively thin for pure Co one (Fig. 12i), with the increase of Co content, the framework of the system transits from a coarser Mn skeleton to a finer Co framework (Fig. 12b~h). C, O, Co and Mn elements uniformly distribute on the skeletons (Fig. 12j), and the content ratio of Mn and Co equals the starting materials as 2:1 (Fig. S4).

Different from Mn and Co, the macrostructure of Zn and Mn binary composite changes with the amount of the two precursor salts, and not all of them can maintain a truly cocontinuous macrostructure, as shown in Fig. 13. When the amount of one precursor is dominant (Fig. 13a,i), there is also a tendency for cocontinuation. However, as the content of the other component increases, the trend of cocontinuation gradually decreases (Fig. 13b,c,h,g). As the content of the other component further increases, the trend of cocontinuation disappears completely and only the porous structure can be observed (Fig. 13d,f). Nakanishi *et al*.^32^ found that when Fe^3+^, Zn^2+^ inorganic salt precursors were used to prepare binary composite macroporous monoliths, because of the large difference of hydrolysis rate constants pKa(Fe^3+^)(2.2) and pKa(Zn^2+^)(9.2), the obtained structure gradually transited from a cocontinuous macroporous structure to the accumulation of particles as binary precursor content increases. The literature^33^ reported that the hydrolysis rate constants of pKa(Mn^2+^), pKa(Fe^2+^), pKa(Co^2+^), pKa(Ni^2+^), and pKa(Zn^2+^) are 10.6, 8.3, 9.8, 8.9 and 9.0, respectively. The difference in hydrolysis rate between Ni^2+^ and Co^2+^, Zn^2+^ and Fe^2+^, Mn^2+^ and Co^2+^ binary is 0.9, 0.7 and 0.8 respectively, and the difference in hydrolysis rate between Zn^2+^ and Mn^2+^ is 1.6. For inorganic salt precursors, the hydrolysis process controls the entire hydrolysis and polycondensation process. If the hydrolysis rate constants of the binary system differ greatly, the element with a faster hydrolysis rate first forms the corresponding products, and the slower one forms after, which leads to a large difference in local structure and composition. This explains from the side that the Zn^2+^ and Mn^2+^ system failed to form a cocontinuous macroporous structure due to the large difference in the hydrolysis rate constants. The macrostructure does not form, while C, O, Zn and Mn elements are evenly distributed on the particles, indicating the uniform composition of the resultant sol and xerogels (Figs. 13j, S5).

Because of a little difference in hydrolysis rate between Ni^2+^ and Co^2+^, Zn^2+^ and Fe^2+^, binary macroporous transition metal composites can be prepared via the sol-gel process accompanied by phase separation. The cooperative control mechanism of sol-gel transition and phase separation of binary Ni-Co system have discussed in our previous works^34,35^, and the resultant unique macrostructure endows excellent performances in battery electrode application^35^. In next work, various single or binary macroporous transition metal hydroxide, oxides and alloy monoliths can be further studied and applied in the fields of electrochemistry, electrocatalysis and chemical adsorption.

## Conclusions

Macroporoustransitional metal hydroxide monoliths were prepared by using sol-gel process accompanied by phase separation with inorganic salt as precursor, PAA as phase separation inducer and PO as gelation promoter in water-glycerol system. PAA can induce the spinodal decomposition to lead to phase separation of the system, and form the framework and combine with Zn hydroxide to inhibit the crystallization of Zn hydroxide. PO controls the sol-gel transition of the system to fix the macrostructure derived from phase separation. The water-glycerol mixed solvent has a great influence on the morphology and macropore size of the monolith. When the appropriate amount of PAA, PO and solvents are added, Zn hydroxide monolith with cocontinuous skeletons and interconnected macropores can be obtained. This approach is also extended to successfully prepare other single transition metal or binary composite transition metal hydroxide monoliths.

## Supplementary information

Supplementary information.
