# Supplementary material for: Preparation of macroporous transition metal hydroxide monoliths via a sol-gel process accompanied by phase separation
Source: Sci Rep. 2020 Mar 9;10:4331. doi: 10.1038/s41598-020-61195-9 (PMC7062902; doi:10.1038/s41598-020-61195-9)
Supplement: Supplementary file 1 — Supplementary information. [file 41598_2020_61195_MOESM1_ESM.pdf]

Supporting Information

Preparation of macroporous transition metal hydroxide monoliths via a sol-gel process accompanied by phase separation

Fu Liu[a], Daoyan Feng[a], Hui Yang[a,b], Xingzhong Guo[a*]

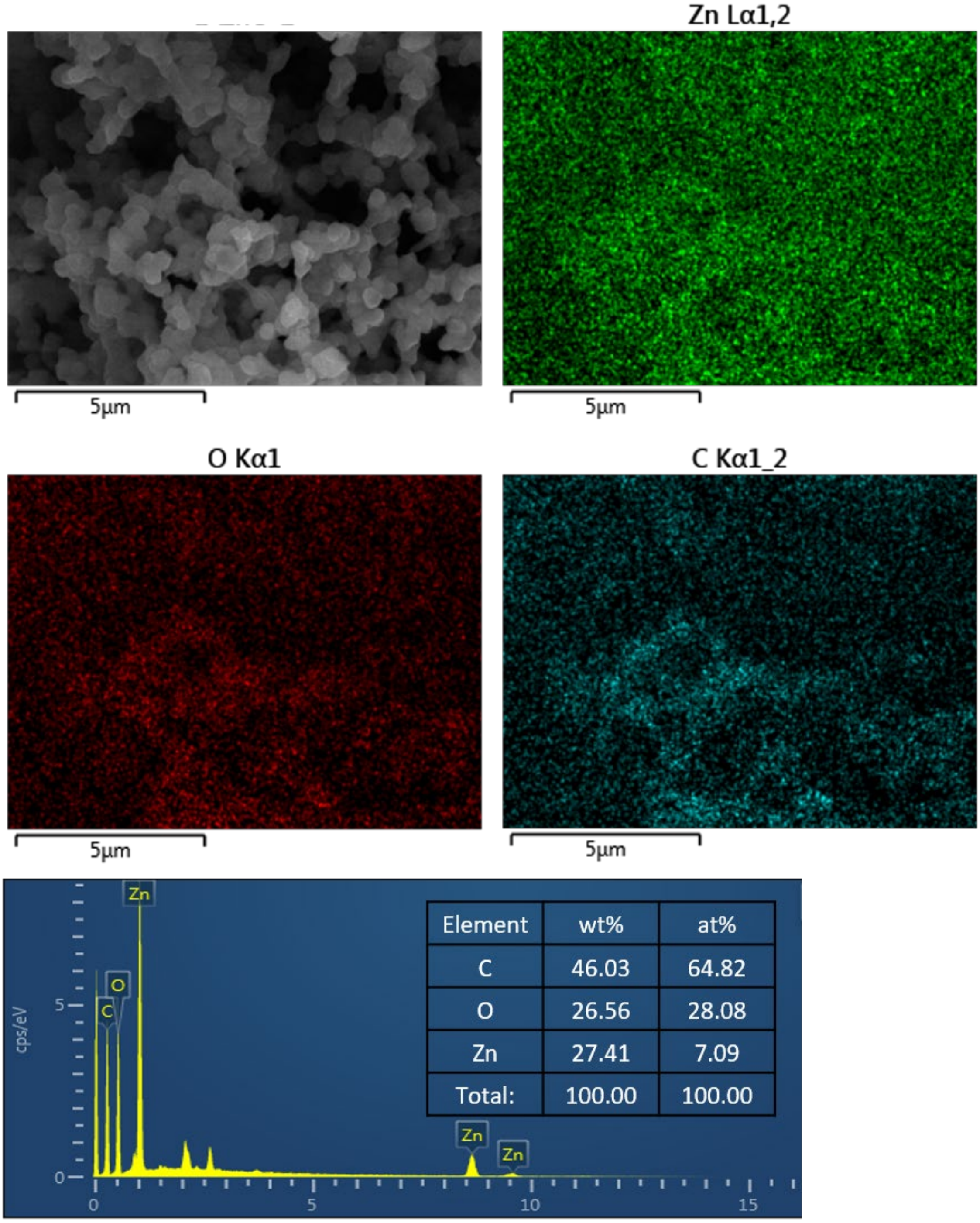

Figure S1 SEM and the corresponding EDS elemental mappings of zinc hydroxide xerogel

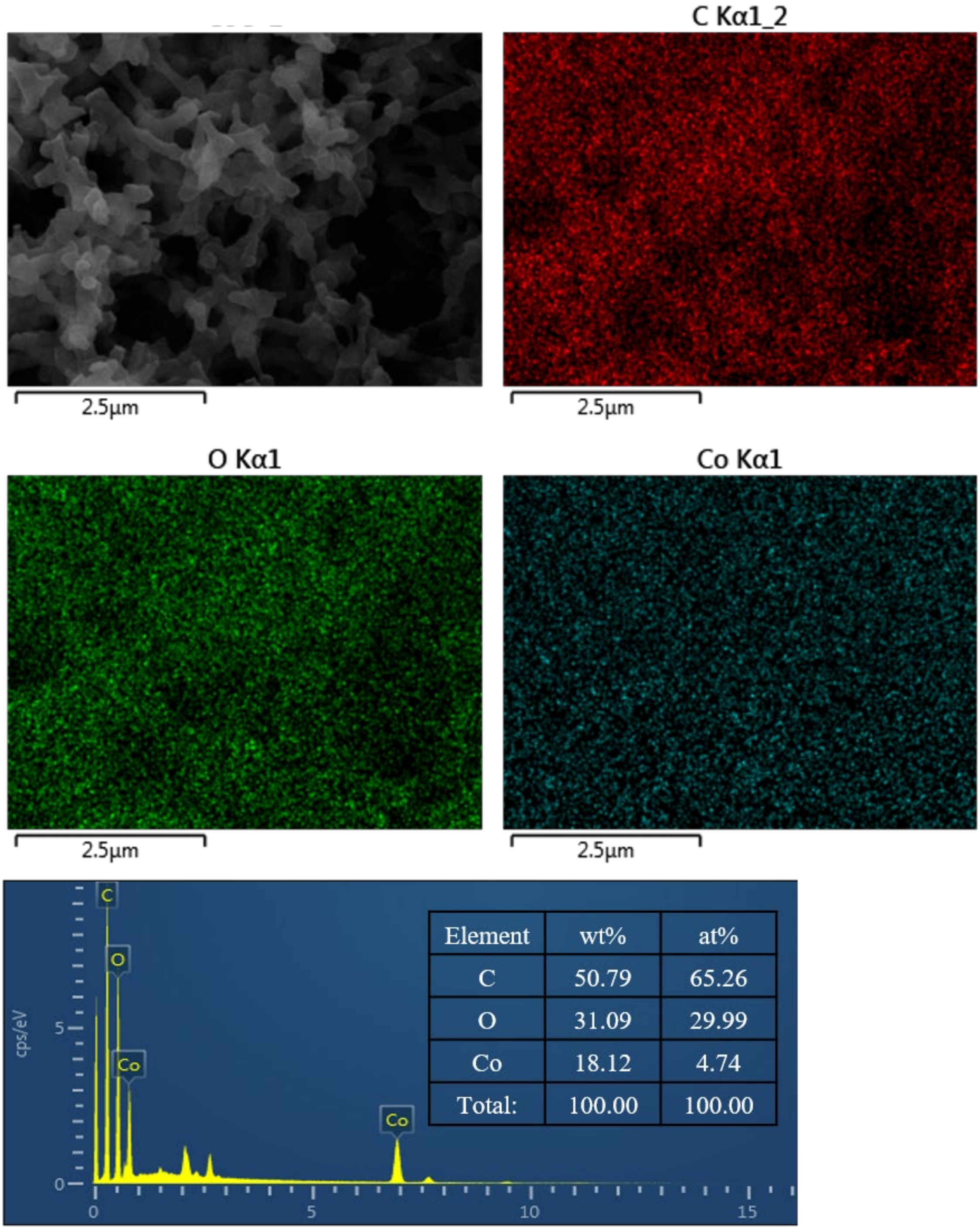

Element	wt%	at%
C	50.79	65.26
O	31.09	29.99
Co	18.12	4.74
Total:	100.00	100.00

Figure S2 SEM and the corresponding EDS elemental mappings of cobalt hydroxide xerogel

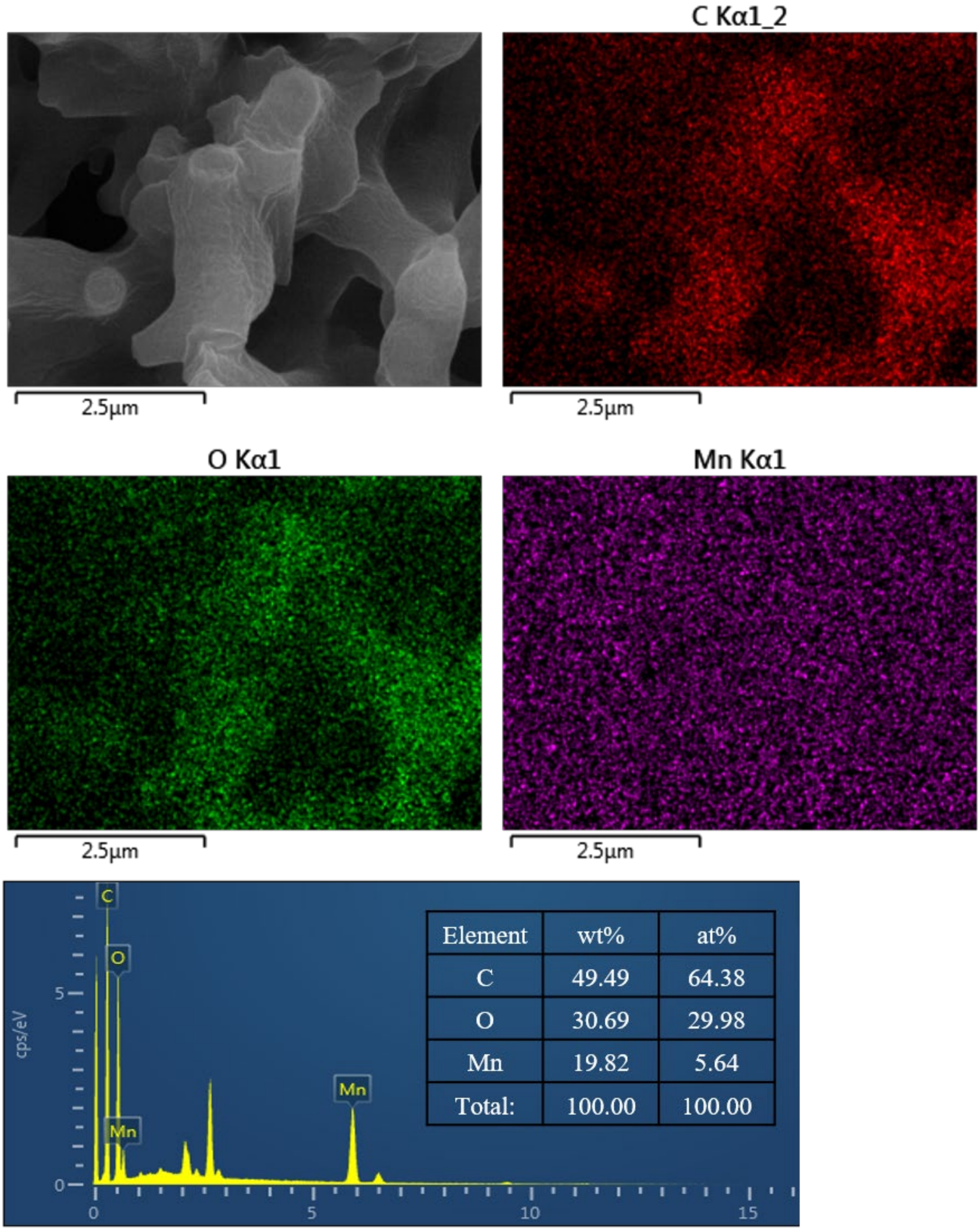

Element	wt%	at%
C	49.49	64.38
O	30.69	29.98
Mn	19.82	5.64
Total:	100.00	100.00

Figure S3 SEM and the corresponding EDS elemental mappings of manganese hydroxide xerogel

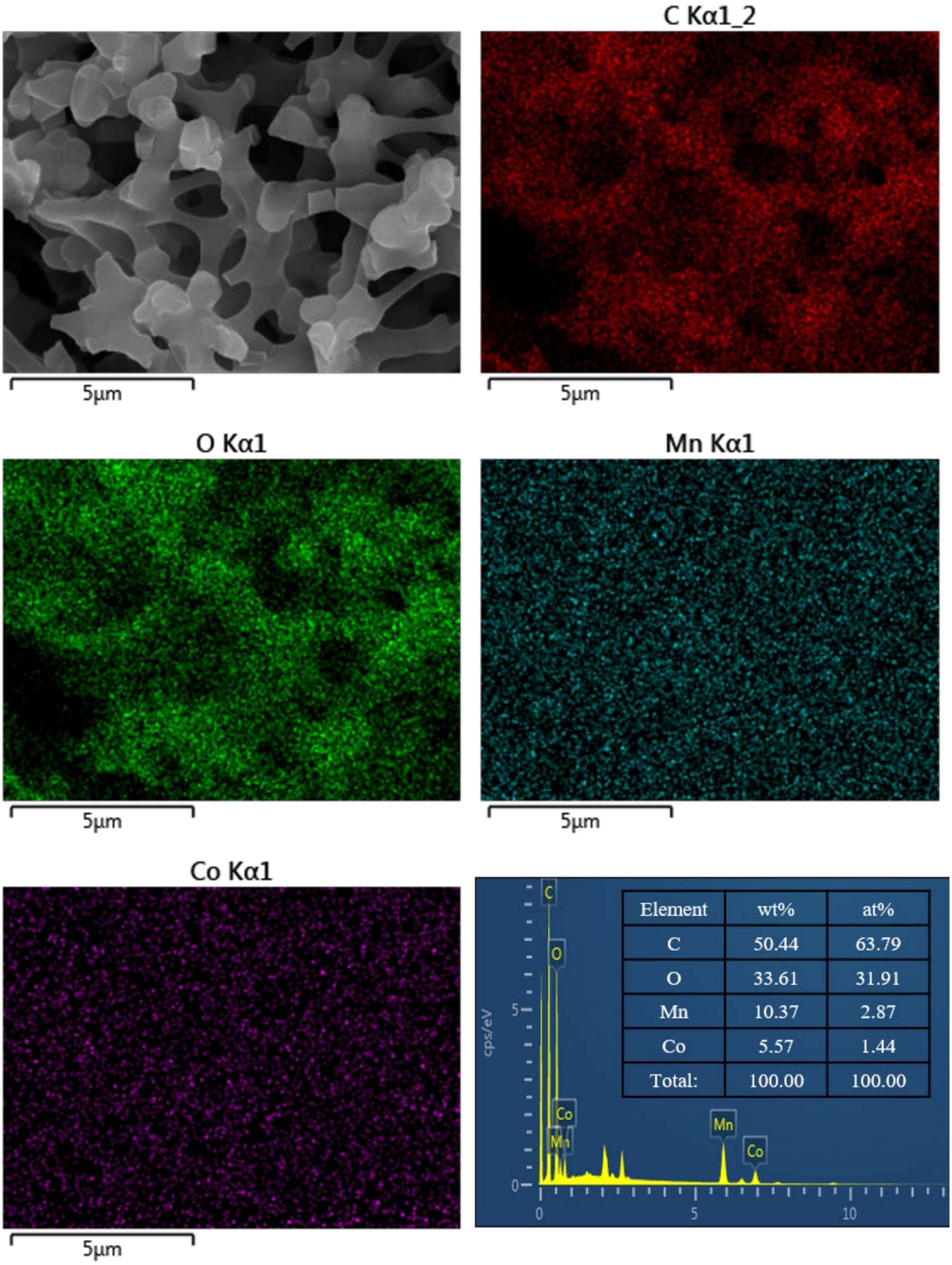

Figure S4 SEM and the corresponding EDS elemental mappings of sample with Mn : Co=2:1.

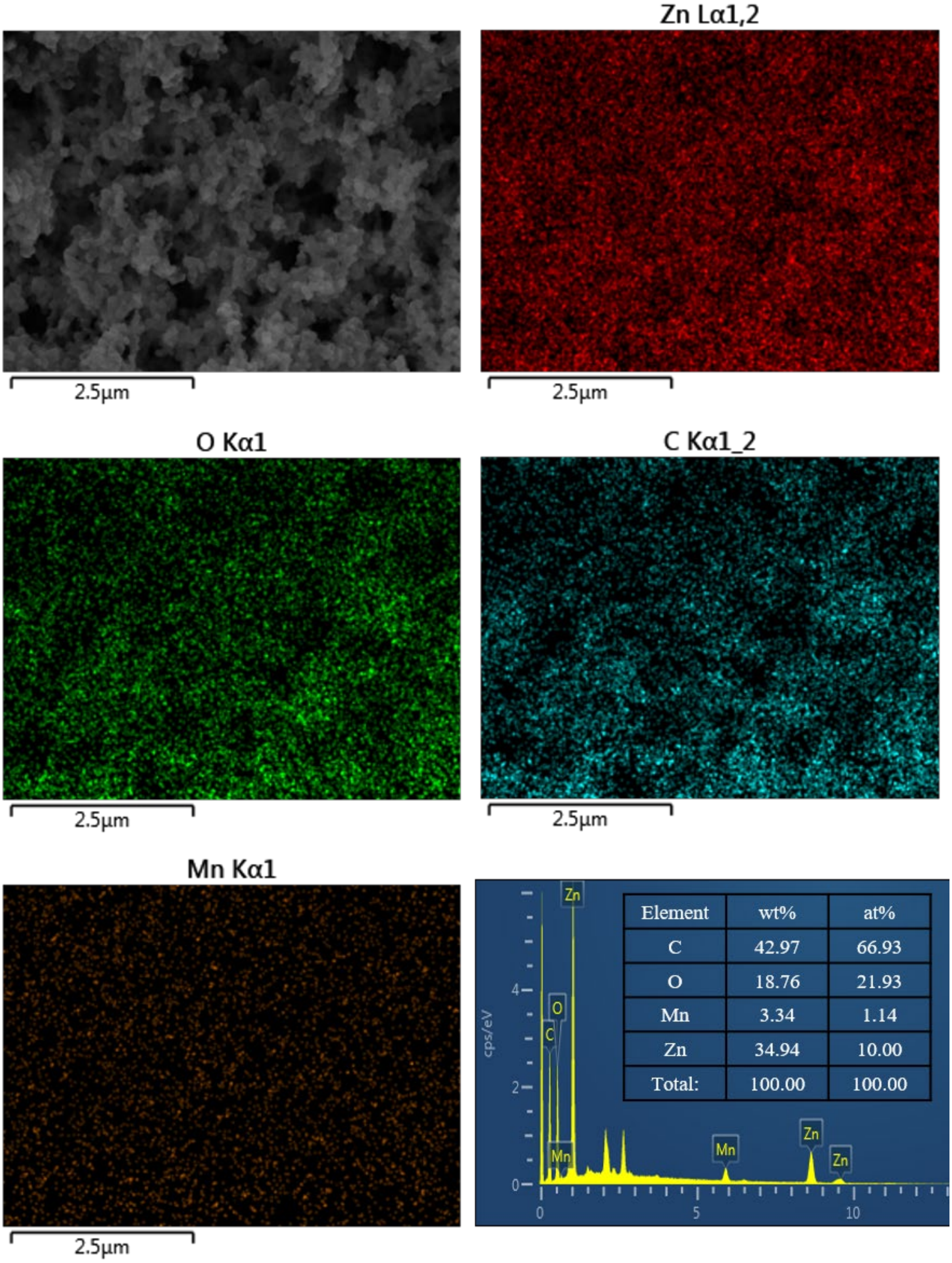

Element	wt%	at%
C	42.97	66.93
O	18.76	21.93
Mn	3.34	1.14
Zn	34.94	10.00
Total:	100.00	100.00

Figure S5 SEM and the corresponding EDS elemental mappings of sample with Zn : Mn=9:1.
